# Apparent coordination of isocitrate dehydrogenase and glutamate decarboxylase expression in early stages of tree development

**DOI:** 10.1186/1753-6561-5-S7-P66

**Published:** 2011-09-13

**Authors:** Juan Jesús Molina-Rueda, María Belén Pascual, Lara Silvia Jiménez-Bermúdez, Francisco Miguel Cánovas, Fernando Gallardo

**Affiliations:** 1 Present address: Rutgers University, Dpt Biological Sciences, Newark, New Jersey, USA; 2 Universidad de Málaga, Dpt. Biología Molecular y Bioquímica, Málaga, Spain; 3 Universidad de Málaga, SCAI, Servicio de Cultivo de Plantas, Málaga, Spain

## 

The biosynthesis of 2-oxoglutarate and glutamate are key steps in the biosynthesis of nitrogen compounds and plant development. The reaction catalyzed by cytosolic isoenzyme of NADP+-linked isocitrate dehydrogenase (IDH) is also considered as the main route in the production of 2-oxoglutarate. According to its expression pattern during development, IDH is also involved in other, yet unknown, processes [1,2]. In addition to the importance of glutamate in the biosynthesis of nitrogen compounds, glutamate also serves as precursor of GABA, a molecule that is currently considered as a signal in higher plants. GABA is produced by the action of glutamate decarboxylase (GAD), a cytosolic enzyme that is regulated by Ca2+/calmodulin and pH. In contrast to IDH, that it is encoded by just one gene in most of plant genomes [2], GAD is encoded by a small family of nuclear genes [3]. The expression of IDH and GAD has been investigated during the differentiation of hypocotyl and stem in tree species. Our results indicate a coordination of the expression of IDH and GAD in developmental processes suggesting a role for 2-oxoglutarate supply and GABA synthesis during early stages of organ differentiation in trees.
